# Non-Genetic Direct Reprogramming and Biomimetic Platforms in a Preliminary Study for Adipose-Derived Stem Cells into Corneal Endothelia-Like Cells

**DOI:** 10.1371/journal.pone.0109856

**Published:** 2014-10-15

**Authors:** Ying Dai, Yonglong Guo, Chan Wang, Qing Liu, Yan Yang, Shanyi Li, Xiaoling Guo, Ruiling Lian, Rongjie Yu, Hongwei Liu, Jiansu Chen

**Affiliations:** 1 Key Laboratory for Regenerative Medicine of Ministry of Education, Jinan University, Guangzhou, China; 2 Ophthalmology Department, First Affiliated Hospital of Jinan University, Guangzhou, China; 3 Institute of Ophthalmology, Medical College, Jinan University, Guangzhou, China; 4 Bioengineering Institute of Jinan University, Guangzhou, China; Instituto Butantan, Brazil

## Abstract

Cell fate and function can be regulated and reprogrammed by intrinsic genetic program, extrinsic factors and niche microenvironment. Direct reprogramming has shown many advantages in the field of cellular reprogramming. Here we tried the possibility to generate corneal endothelia (CE) -like cells from human adipose-derived stem cells (ADSCs) by the non-genetic direct reprogramming of recombinant cell-penetrating proteins Oct4/Klf4/Sox2 (PTD-OKS) and small molecules (purmorphamine, RG108 and other reprogramming chemical reagents), as well as biomimetic platforms of simulate microgravity (SMG) bioreactor. Co-cultured with corneal cells and decellularized corneal ECM, Reprogrammed ADSCs revealed spherical growth and positively expressing Nanog for RT-PCR analysis and CD34 for immunofluorescence staining after 7 days-treatment of both purmorphamine and PTD-OKS (P-OKS) and in SMG culture. ADSCs changed to CEC polygonal morphology from spindle shape after the sequential non-genetic direct reprogramming and biomimetic platforms. At the same time, induced cells converted to weakly express CD31, AQP-1 and ZO-1. These findings demonstrated that the treatments were able to promote the stem-cell reprogramming for human ADSCs. Our study also indicates for the first time that SMG rotary cell culture system can be used as a non-genetic means to promote direct reprogramming. Our methods of reprogramming provide an alternative strategy for engineering patient-specific multipotent cells for cellular plasticity research and future autologous CEC replacement therapy that avoids complications associated with the use of human pluripotent stem cells.

## Introduction

An important breakthrough was reported by Yamanaka and colleagues who succeeded in directly reprogramming fibroblasts into induced pluripotent stem cells (iPSCs) by transduction of the four transcription factors of Oct4, Sox2, Klf4 and c-Myc in 2006 [1]. Such somatic cell reprogramming into pluripotency based iPSC factors has made a lot of achievements, which can provide many insights about cellular plasticity [2]. Reprogramming of iPSCs can be achieved by influencing the epigenetics and key signaling pathways with small molecules. For example, in combination with only Oct4 factor, the activation of sonic hedgehog signaling (such as purmorphamine) could reprogram mouse fibroblasts into iPSCs [3]. However, direct differentiation of cells from a pluripotent state is always complicated and time consuming with potential safety concerns. Lately, it has been found that direct conversion between different somatic cell lineages (also called as direct reprogramming) offers benefits of higher efficiencies and shorter times [4]. Recent studies also indicated that direct reprogramming of cells by which differentiated cell may convert into another cell-type could be realized by transitioning through unstable plastic intermediate states. This process is generally associated with an initial epigenetic erasure phase achieved by iPSC-factor-based somatic cell reprogramming and subsequent differentiation by exposure to developmental and other signal cues [5]–[7]. Szabo et al. demonstrated the ability of human fibroblasts to be directly converted to multipotent haematopoietic progenitors of the myeloid, erythroid and megakaryocytic lineages via the use of Oct4 together with haematopoiesis promoting conditions [8]. Kim et al. reported the generation of neural stem/progenitor cells (NPCs) from mouse fibroblasts by transient expression of the four iPSC-factors within 9–13 days [9].

However, the majority of published direct reprogramming protocols relies on viruses, which may raise safety issues and preclude their clinical use [5], [10]. If above direct reprogramming processes can be manipulated using exogene-free methods such as protein transduction and small molecules, it could form safe and convenient cell reprogramming like the generation of protein iPSCs (piPSCs) or chemically iPSCs (CiPSCs) [11]–[15]. Reprogramming proteins can be delivered into cells both in vivo and in vitro when they are fused in frame to protein transduction domains (PTD). NPCs derived from human piPSCs and embryonic stem cells (ESCs) were highly expandable without senescence while NPCs from virus-based hiPSCs showed limited expandability and early senescence [16]. CiPSCs utilize the chemical reprogramming strategy via small molecules which have many advantages such as safer, faster, reversible, non-immunogenic and controllable. Specific combination of small molecules was a promising approach for manipulation of cell reprogramming and plasticity [15], [17]. The combined treatment with both reprogramming proteins and small molecules displayed higher efficiency and better results [13], [18]. It was reported that epigenetic modulators of histone deacetylase inhibitor trichostatin A (TSA) and DNA methyltransferase inhibitor RG-108 together with reprogramming proteins of Oct4/Klf4/Sox2 could activate and maintain pluripotent state in NPCs. None of the factors of the combination alone was sufficient to reprogram neural stem cells into a stable pluripotency state [19].

The fate and function of stem cells are regulated by both intrinsic genetic program and niche microenvironment. The “biomimetic’’ environments can direct the changes of stem cells. Biomimetic platforms in vitro include regulatory molecules and signals from culture condition and other cells (e.g., co-culture) [20], extracellular matrix (ECM) environment (e.g., decellularized ECM scaffold) [21], [22], and physical factors (e.g., bioreactor dynamic condition) [23], [24], which can be established to act in concert, with synergistic and competing effects on the reprogramming and differentiation of stem cells [25]. We previously found that 1/4 suspension of iPSCs labeled with 10 nmol/L quantum dots and 60% confluence of rabbit corneal endothelial cells (CECs) showed optimal effects on mixture co-culture each other and cell labeling. iPSCs morphologically changed to endothelial-like cells after mixed culture with rabbit CECs and expressed aquaporin1 (AQP1) of CECs marker by immunofluorescence stain [26]. Our previous studies also revealed that rabbit corneal stromal cells (CSCs) on the scaffolds of decellularized bovine cornea ECM under simulate microgravity (SMG) rotary cell culture system (RCCS) tended to spherical aggregate growth, while they only showed monolayer two-dimensional (2-D) growth in static culture [27]. Rabbit CSCs in SMG showed round shape with many prominences and were more prone to grow into the pores of decellularized cornea ECM with aggregation when supplemented with valproic acid (VPA), vitamin C (VC) and 10% fetal bovine serum (FBS). However, rabbit CSCs in plastic just displayed spindle shape and rare interconnected [28].

In this study, we investigate the effects of recombinant cell-penetrating reprogramming proteins Oct4/Klf4/Sox2 (PTD-OKS), small molecules (RG108, Reprogramming Cocktail Set I and purmorphamine) and SMG bioreactor on the reprogramming of human adipose-derived stem cells (ADSCs), as well as their preliminary commitment into corneal endothelia-like cells by co-cultured with corneal cells and seeded on decellularized corneal ECM. The goal was to understand if the combination of PTD-OKS proteins, small molecules and biomimetic environments (e.g., bioreactor, co-culture and decellularized ECM scaffold) was able to act in synergistic concert and be used as a suitable platform for non-genetic direct reprogramming of ADSCs into corneal endothelia-like cells.

### Ethics Statement

Six women with a mean age of 35.1±6.5 years were selected for the study after written informed consent was obtained. The institutional ethical review board of the First Affiliated Hospital of Jinan University approved the protocols. A tumescent solution consisting of a mixture of 0.9 NaCl, 0.1% lidocaine, and 1∶100000 epinephrine was injected using a 50 ml syringe into the fat donor sites of each patient's abdomen. A 2.5-mm-diameter cannula and 20-ml-syringes were used to harvest 200 ml adipose tissue from left abdomen of each patient. All the data used in this study was anonymized.

Primary cultures were established from the corneas of New Zealand White rabbit (4 eyes) which were aged 3–4 months old with a weight range of 2–2.5 kg from Guangdong medical laboratory animal center. First, rabbits were fed with basic feed in single cage at 20–26°C and 40–70% relative humidity condition. The rabbit was sacrificed using 80∼100 ml/kg over dose of sodium pentobarbital injected into the ear vein rapidly under supervision of vet, eyes were obtained and corneas were excised. Rabbits were handled in accordance with the ARVO Statement on the Use of Animals in Ophthalmic and Vision Research. The protocol was approved by the Institute Animal Care and Use Committee of Jinan University (Permit Number: 20130902001). The bovine eyes were obtained at a local slaughter house (Jiang Village, Duo Ying Poultry Co. Ltd., Guangzhou, Guangdong, China) and their corneas were checked to be free of defects by slit lamp examination and processed as previous way [28].

## Materials and Methods

### Materials

Culture reagents were from Gibco (Grand Island, NY, USA). ADSCs were cultured in a conventional medium that consisted of Dulbecco’s Modified Eagle’s Medium (DMEM), supplemented with 100-U/mL penicillin G sodium, 100-mg/mL streptomycin sulfate, and 10% vol/vol FBS. Unless otherwise stated, all the other reagents were from Sigma (St. Louis, MO, USA). VPA was from Suju (Guangzhou, China). RG108, Reprogramming Cocktail Set I and purmorphamine were from Biovision (San Francisco, USA). Cell Counting Kit-8 (CCK-8) was from Dojindo (Kyushu, Japan). Cell Cycle and Apoptosis Analysis Kit and Annexin V-FITC/PI apoptosis detection kit were from KeyGEN (Nanjing, China). Monoclonal anti-vimentin (NeoMarkers) was from Lab Vision Corp (Fremont, MO, USA). Goat anti-CD34 polyclonal antibody, goat anti-mouse IgG and goat anti-rabbit IgG were from Santa Cruz Biotechnology (Santa Cruz, CA, USA). EZgeneTM Tissue RNA Miniprep Kit was from Biomiga (San Diego, CA, USA). ReverTra Ace qPCR RT Kit, Blend Taq and Blend Taq-Plus were from Toyobo (Osaka, Japan). Primers were synthetized by BGI (Shenzhen, China).

### Preparation and activity identification of reprogramming proteins

The reprogramming proteins including Oct4, Klf4 and Sox2 were expressed and purified as fusion proteins with an N-terminally linked protein transduction domain (PTD) of amino acid sequence YGRKKRRQRRR and 6-His purification tag at the C-terminal respectively. PTD used here is an 11-amino acid cell penetrating peptide derived from the human immunodeficiency virus type 1 (HIV) Tat protein. The plasmids containing the Oct4, Klf4 and Sox2 gene strains pCX-OKS-2A were obtained from Addgene (http://www.addgene.org). E. coli strain ER2566 and prokaryotic expression plasmid pKYB were purchased from New England Biolabs (NEB). In brief, the gene encoding the fusion proteins were cloned into the expression vector pKYB to construct the recombinant expression vectors. After the recombinant vectors were transformed into the *Ecoli.* strain ER2566, the fusion proteins such as PTD-Oct4, PTD-Klf4 and PTD-Sox2 were expressed and purified by Ni-affinity chromatography. The binding activities of the recombinant reprogramming proteins with their target sequences were identified using fluorescence resonance energy transfer (FRET) assays as mentioned before [29]–[31]. Briefly, two single-stranded oligonucleotides of sequences of Oct4, Klf4 and Sox2 were produced by chemical synthesis, which connected anthocyan dye (CY3) (excitation wavelength of excitation of 550 nm, emission wavelength of emission of 575 nm) at the 5′ end. The specific sequences of Oct4, Klf4 and Sox2 were shown in table 1. Each double strands DNA sequence was obtained by annealing of two reverse compliment single DNA strand, which was synthesized by Invitrogen (Guangzhou, China). Cy3-labeled double-stranded target DNA sequences specific binding Oct4, Klf4 and Sox2 were obtained by denaturing annealing (95°C 5 min, 37°C 2 min, 0°C 2 min). The recombinant proteins of PTD-Oct4, PTD-Klf4 and PTD-Sox2 were labeled with isothiocyanate fluorescein FITC (excitation 490 nm, emission 525 nm) using FITC labeling kit (Xirun. Bio. China). The binding of the reprogramming proteins of PTD-Oct4, PTD-Klf4 and PTD-Sox2 with their target sequences of Oct4, Klf4 and Sox resulted in the energy transferring from FITC to Cy3. The fluorescence emission energy scanning from FITC labeled reprogramming proteins following the addition of its Cy3 labeled target DNA sequences was performed on a multiple function scanner (Perkin Elmer, German) using an non-target DNA sequence as negative control. And the variation of the emission spectrum was detected to confirm the fluorescence resonance energy transferring which represented the binding of the recombinant reprogramming proteins with their target sequences.

**Table 1:** List of the specific sequences of Oct4, Klf4 and Sox2.

Oct4-F: 5′-cy3-ATGCATGCAAATATGCAAAT-3′
Oct4-R: 5′-cy3-CAGT ATTTGCATATTTGCAT-3′
Klf4-F: 5′-cy3-ATGCACCCCAGTCACCCTAGC-3′
Klf4-R: 5′-cy3-TCTAGGGTGATAGGGTGCAT-3′
Sox2-F: 5′-cy3-CAGTCAAACAAAGACAAACAAAGAGCAT-3′
Sox2-R: 5′-cy3-ATGCACTTTGTTTGTCTTTGTTTGACTG-3′

### The culture of rabbit corneal cells and the preparation of decellularized corneal ECM

The isolation and culture of rabbit CECs and CSCs: Eyes from New Zealand White rabbits were obtained and cornea was excised. Connective tissue and external muscles were then removed. The corneas were rinsed with saline containing antibiotic solution. Descemet’s membrane with intact endothelial cells was meticulously dissected from corneas and placed in a culture dish containing 0.25% trypsin solution for 10–20 seconds, then washed in culture medium. Rabbit CECs were centrifuged (1600 rpm, 5 min), and suspended at a concentration of 5×10^5^ cells/mL in culture medium. The corneas stripped of both endothelial and epithelial cells were placed in a solution of 2.0 mg/mL collagenase I in culture medium overnight at 37°C. Rabbit CSCs were washed in culture medium, centrifuged (1600 rpm, 5 min), and suspended at a concentration of 5×10^5^ cells/mL in culture medium. The cells were seeded onto culture plates.

The preparation of B-ECM: The primary bovine CECs were seeded into six-well at 5×10^3^ density, fed with 2 mL of medium, and incubated at 37°C in a 5% CO_2_ incubator. When the cells reached 60–70% confluence, the medium was changed into conventional DMEM medium containing 4% (w/v) dextran T-40 for 7 days. 1–8 ng/ml basic fibroblast growth factor (bFGF) was added every other day. Last, culture medium was aspirated and added 0.5% Triton X-100 and 20 mM NH_4_OH solution for 3–5 min until cells detached and washed with phosphate buffered saline (PBS).

The preparation of decellularized cornea: Fresh bovine eyes were obtained and the cornea was excised, rinsed with saline containing antibiotic solution (prepared with 100-U/mL penicillin G sodium and 100-mg/mL streptomycin sulfate), and dissected under sterile condition. Bovine stromal lamella (1 mm thick) was removed, treated with 0.5% Triton X-100 and 20 mM NH_4_OH mixture for 5–10 min. After rinsed with PBS three times, bovine stromal lamellas were frozen in −80°C for 3 d and then preserved in 100% glycerol at 4°C. Prior to use, the dehydrated bovine stroma was rehydrated in PBS. Then, the stroma was cut into pieces and sterilized under ultraviolet light for 30 min.

### The isolation and primary culture of ADSCs

Adipose tissue was repeatedly washed with PBS until blood was completely removed from the tissue, and then incubated with equal volume of DMEM containing 0.1% type I collagenase at 37°C for 1 h in a shaking incubator at 110 rpm. The suspension was filtered through 100 µ nylon membrane and centrifuged (300 g, 10 min). The ADSCs were then rinsed in the culture medium composed of DMEM, centrifuged (300 g, 5 min), and suspended at a concentration of 1×10^4^ cells/mL in a conventional medium supplemented with 3.7 g/L NaHCO_3_, 100-U/mL penicillin G sodium, 100-mg/mL streptomycin sulfate, and 10% (vol/vol) FBS. The cells were seeded into a 25 cm^2^ plastic culture flask, fed with 4 mL of medium, and incubated at 37°C in a 5% CO_2_ incubator. The culture medium was changed every second day.

### Surface phenotypes of human ADSCs

In order to characterize the phenotype of expanded ADSCs, cells at passaged-1(P1) were detached by 0.25% trypsin-EDTA and after suspension in 100 ml of PBS. Then cells were separately incubated with the following antibodies in the dark at 48°C for 30 min. CD29, CD44, CD59, CD45, HLA-DR, CD105 and CD34 were conjugated with fluorescein isothiocyanate (all from BD Biosciences). Next, cells were washed three times before they were analyzed by a FACS Calibur flow cytometer (Becton Dickinson, Franklin Lakes, NJ, USA).

### Osteogenic and adipogenic differentiation of human ADSCs

ADSCs were plated at 1×10^4^ cells/mL and cultured in conventional medium for 24 h. Afterward, the medium was changed to an adipogenic induction medium (DMEM/F12, 10% FBS, 1 µM insulin, 200 µM indometacin, 1 µM dexamethasone, 0.5 mM isobutyl-methylxanthine and 1% antibiotic-antimycotic). The medium changed every 3 days until 2 weeks. Adipogenic differentiation was confirmed by staining of lipids with Oil red O.

Cells were plated at 1×10^4^ cells/mL and cultured in normal medium for 24 h. Afterward, the medium was changed to an osteogenic induction medium (DMEM/F12, 10% FBS, 0.1 µM dexamethasone, and 50 mg/l 2-phosphate ascorbic acid, 10 mM β-glycerophosphate, 1% antibiotic -antimycotic). The medium changed every 3 days until 2 weeks. Osteogenic differentiation was confirmed by staining calcium-rich deposits with Alizarin red.

### The transduction of reprogramming proteins into ADSCs

Human ADSCs at 1×10^4^ cells/mL were seeded in 24-well plates and cultured in conventional medium overnight. Next day the culture medium was removed and the cells were washed with PBS. 8 µg/ml recombinant reprogramming proteins of serum-free medium were respectively added to ADSCs. Cells were incubated in PTD-Oct4, PTD-Klf4 or PTD-Sox2 for 4 h at 37°C, and ADSCs incubated with PBS for 4 h were used as negative control. Then cells were washed with PBS. ADSC conventional medium was added and ADSCs were incubated for 20 h. Cells were examined by immunofluorescence staining using specific antibodies of Oct4, Klf4 and Sox2.

### Non-genetic ADSCs direct reprogramming platform through reprogramming proteins, small molecules and SMG bioreactor

#### Primary test of reprogramming reagents of PTD-OKS reprogramming proteins and small molecules on human ADSCs

The survival assay and primary morphologic changes of human ADSCs treated with reprogramming reagents were first determined. The groups were prepared as follows:

PTD-OKS proteins (group A): ADSCs were plated at 1×10^4^ cells/mL and cultured in induction medium [DMEM/F12, 7.5% KSR, 2.5% FBS, 2.5% Albumax II (200 mg/mL), 0.1 mM non-necessary amino acid (NEAA), 0.5% insulin-transferrin-selenium(ITS), 1 mM sodium pyruvate, 2 mM L- glutamine, 100 µM β- mercaptoethanol, 5 ng/mL bFGF] supplemented protein PTD-OKS (8 µg/ml) for 16 h, then the medium changed into conventional medium for another 8 h. Cells were detected after 7 cycle treatments.

PTD-OKS proteins supplemented with RG108 and other small molecules (group B): ADSCs were plated at 1×10^4^ cells/mL and cultured in induction medium supplemented 100 nm RG108 and Reprogramming Cocktail Set I (containing 0.5 uM A83-1, 3.0 uM CHIR99021, 0.5 uM PD0325901 and 0.5 uM Thiazovivin) overnight. Then cells cultured in conventional medium for 8 h. After these small molecules and PTD-OKS (8 µg/ml) were added overnight, ADSCs cultured in the conventional medium for 8 h. Cells were detected after several cycle treatments until cell aggregates occurred.

PTD-OKS proteins supplemented with purmorphamine (group C): ADSCs were plated at 1×10^4^ cells/mL and cultured in induction medium supplemented 1 mM purmorphamine overnight. Then cells cultured in conventional medium for 8 h. After PTD-OKS (8 µg/ml) and purmorphamine were added overnight, ADSCs cultured in the conventional medium for another 8 h. Cells were detected after 7 cycle treatments.

#### ADSCs treated with modified reprogramming reagents and SMG culture

In order to obtain better reprogramming effect on ADSCs, modified reagents and SMG culture were then tried. The groups were as follows:

Modified PTD-OKS proteins supplemented with purmorphamine (group D): The experiment showed that group C displayed better results than group B. According to the observation of primary experiment, later modified procedure for non-genetic ADSCs direct reprogramming was used as follow: primary ADSCs were plated at 1×10^4^ cells/mL and cultured in the conventional medium supplement 1 mM purmorphamine for 2 days. ADSCs cultured in medium 1 (DMEM/F12, 20% KSR, 0.5% ITS, 0.1 mM non-necessary amino acid, 1 mM sodium pyruvate, 2 mM L-glutamine, 100 µM β-mercaptoethanol, 5 ng/mL bFGF and 2% sucrose) supplemented with PTD-OKS (8 µg/ml) and 1 mM purmorphamine for 16 h. Then cells cultured in medium 1 without PTD-OKS and purmorphamine for 8 h. Cells were detected after 7 cycle treatments.

Modified PTD-OKS proteins supplemented with purmorphamine in SMG culture (group E): In SMG experiment group, primary ADSCs were plated in 6-well plate at density of 1×10^4^ cells/mL in the conventional medium supplement 1 mM purmorphamine and incubated at 37°C in a 5% CO_2_ environment for for 2 days, then the culture medium was changed to medium 1 supplemented with PTD-OKS (8 µg/ml) and 1 mM purmorphamine for 16 h. Later the cells cultured with medium 1 without PTD-OKS and purmorphamine for 8 h. Cells were treated for 7 cycle treatments. Then the cells were trypsinized and cultured in medium 1 supplemented with PTD-OKS (8 µg/ml) and 1 mM purmorphamine in the simulated microgravity system as mentioned before [28]. Gas bubbles in the RCCS vessel must be removed. The vessel was put into the incubator and rotational speed was set at 15 rpm. After SMG culture of 5 days, the cells were transferred to a 6-well plate cultured in conventional medium.

**Figure 1:**
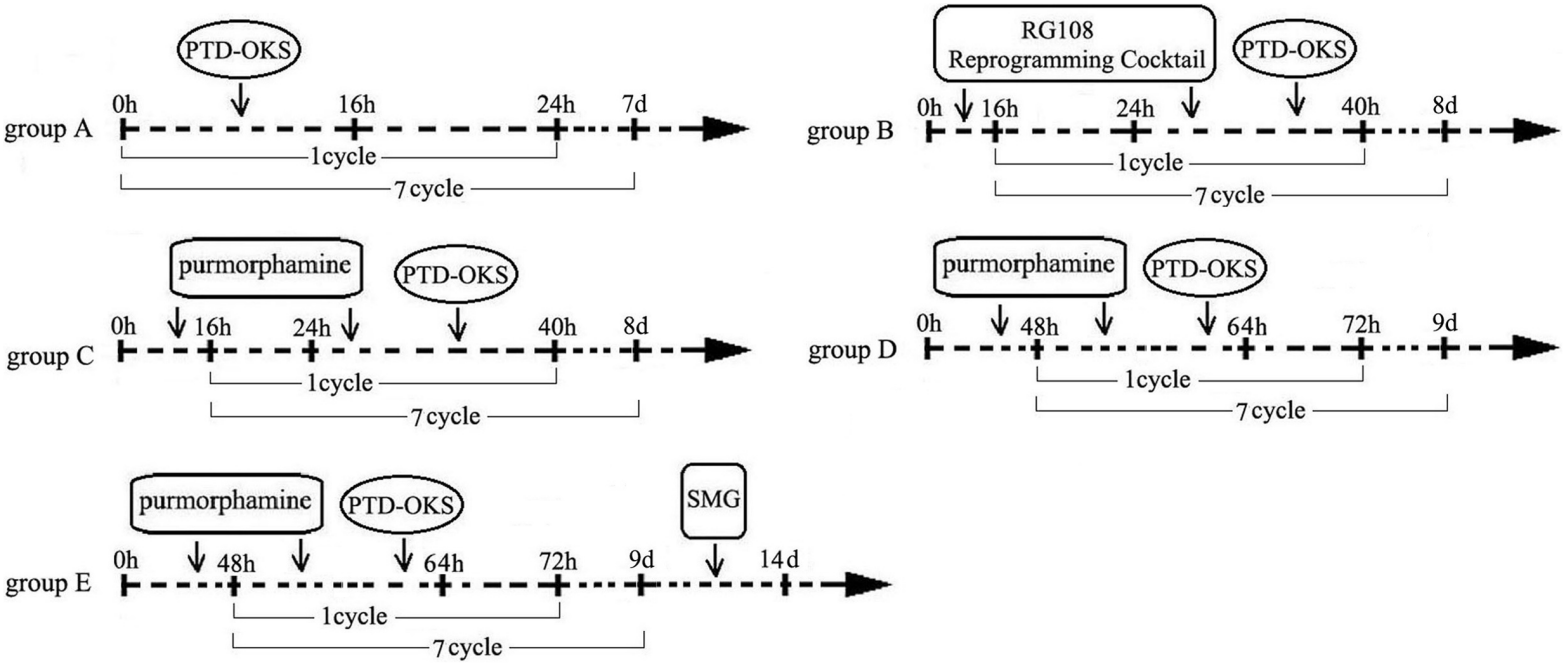
The schematic illustration of reprogramming groups and processes. The schematic illustrated the groups and the processes of sequential non-genetic direct reprogramming platform through reprogramming proteins, small molecules and SMG bioreactor.

Control group: synchronous cultured ADSCs in a conventional medium were used as control group. The schematic illustration of reprogramming groups and processes was shown in Fig. 1.

### Biomimetic platform for the derivation of CEC-like committed cells from reprogrammed ADSCs

#### ADSCs cultured on the B-ECM

After the treatment of reprogramming proteins and small molecules in group D, ADSCs were digested using 0.25% trypsin for 1 min, collection of cells and centrifugation (1600 rpm, 5 min), cells re-plated into the original culture plate containing medium 1 cultured for a week. Then, gentle pipetting after trypsin treatment disaggregated ADSCs clumps into single cells. The cells were seeded onto B-ECM plates and cultured in medium 1 for a week.

#### ADSCs co-culture with corneal cells of CECs and CSCs

The primary rabbit CSCs were digested using 0.25% trypsin for 5 min, collected and centrifuged. The cells suspended with conventional medium, then seeded on the invert of the insert culture plate at 1×10^5^ cells/mL, cultured in 37°C, 5% CO_2_ incubator for 4 h. Rabbit CECs were digested using 0.25% trypsin for 5 min, collected and centrifuged. The cells suspended with conventional medium, and seeded on the inside of the insert culture plate at 5×10^5^ cells/mL for 24 h. Then CECs were treated with 10 µg/ml mitomycin C (MMC) for 3 h, and washed away MMC with PBS for three times. The treated ADSCs were seeded on the inside of the insert and mixed culture with CEC in medium 2 (DMEM/F12, 10% FBS, 0.1 mM non-necessary amino acid, 1 mM sodium pyruvate, 2 mM L-glutamine, 100 µM β- mercaptoethanol, 5 ng/mL bFGF and 1% P/S) at a cell proportion of 1∶1 for 10 days.

#### ADSCs cultured on the decellularized bovine cornea

After co-culture with CECs and CSCs, ADSCs were digested using 0.25% trypsin for 5 min, collected and centrifuged. The cells cultured on the decellularized bovine corneal stroma and culture in the medium 3 (DMEM/F12, 1 µM retinoic acid, 1% FBS, 0.1 µM insulin, 1 mM sodium pyruvate, 0.1 mM non-necessary amino acid, MEM essential vitamins and 1% P/S) and supplemented with GSK-3β inhibitors for 1 week, and then the cells were detected.

### Cell proliferation assay

Cell Counting Kit-8 (CCK-8) was employed to identify the effect of PTD-OKS and small molecules on the proliferation of ADSCs in group A, group B, group C and control group. 1×10^4^ cells/mL were seeded and cultured at 37°C for 24 h, Then the conventional medium was removed. Subsequently, cells were treated with or without PTD-OKS and small molecules, in the presence of 10% FBS for a further 72 h. After 10 ul dye was add to each well, cells were incubated at 37°C for 2 h. The absorbance at 450 nm was determined using multimode reader. Six parallel experiments in each sample were used to assess the cell proliferation.

### Flow cytometry for cell survival assay

To determine the cell cycle of ADSCs within the culture solution with or without PTD-OKS and small molecules, flow cytometry was performed. ADSCs were cultured in medium in 6-well culture plates with or without PTD-OKS and small molecules for 3 days. Cells from the four groups were resuspended in cold alcohol and then stored at −4°C overnight before flow cytometry analysis. Data analysis was conducted using ModFit software. Flow cytometry assay was used to test whether PTD-OKS and small molecules had an apoptotic effect in cultivated ADSCs. The method was performed as manufacture instructions and as previously described [28]. ADSCs were cultured in medium in 6-well culture plates with or without PTD-OKS and small molecules for 3 days till cells were about 60–70% confluent. Cells were harvested and washed in cold PBS. ADSCs were treated with Annexin V-FITC/PI and then resuspended in binding buffer at a concentration of 10^6^ cells/ml. Data analysis was conducted using WinMDI software.

### Reverse transcription–polymerase chain reaction (RT-PCR) analysis

Total RNA from ADSCs was isolated using Tissue RNA Miniprep Kit, and the resulting RNA samples were quantified by measuring the OD at 260 nm; the OD 260/280 ratios for all RNA samples were between 1.8 and 2.1. Total RNA (1 µg) was reverse transcribed in a 10 µl reaction mixture containing 2 µl 5× RT Buffer, 0.5 µl RT Enzyme Mix, 0.5 µl Primer Mix, 6 µl nuclease-free water at 42°C for 1 h. One tenth of the RT product was used for subsequent PCR with the final concentration of PCR reaction being 1× Buffer, 0.2 mM dNTPs, 1.25 U Blend Taq in a total volume of 50 µL, using primers shown in Table 2. The PCR mixture was first denatured at 94°C for 2 min then amplified for 30 cycles (94°C, 30 sec; Tm-5°C, 30 sec; 72°C, 1 min) using an authorized thermal cycler (Eppendorf, Hamburg, GER). After amplification, 5 uL of each PCR product and 1 µl of 6× loading buffer were mixed and electrophoresed on a 1.5% agarose gel in 0.5× Tris-boric acid-EDTA containing 0.5 µg/mL ethidium bromide. Gels were photographed and scanned.

**Table 2:** List of primers.

Primers		Sequences (5′to 3′)	Size (bp)	GeneBank Accession Number
GAPDH	Sense	GGTCGGAGTCAACGGATTTG	219	BC059110
GAPDH	Antisense	TGGAAGATGGTGATGGGATT		
Oct4	Sense	GGGGTTCTATTTGGGAAGGTAT	215	NC_000006
Oct4	Antisense	CCTCTCACTCGGTTCTCGATAC		
Sox2	Sense	CGCATGGACAGTTACGCGCACA	275	NM_003106.
Sox2	Antisense	TCGGACTTGACCACCGAACCCA		
Klf4	Sense	CACCTGCAGCTTCACCTATCCGA	260	HF546201
Klf4	Antisense	CCTTCAGCACGAACTTGCCCAT		
Nanog	Sense	CCCCAGCCTCTACTCTTCCTAC	268	NC_000001
Nanog	Antisense	CAAGTCACTGGCAGGAGAATTT		

### Immunofluorescence assay

Immunofluorescence was used to identify the ADSCs. Briefly, after fixation in 4% paraformaldehyde for 30 min at room temperature, ADSCs were permeabilized with 0.1% Triton X-100 in PBS for 15 min at room temperature, washed three times with PBS and incubated with PBS containing 10% FBS for 30 min at room temperature. The cells were incubated with the monoclonal 500 µl Anti-Vimentin (1∶500 dilution), Anti-CD34 (1∶400 dilution), Anti-Oct4 (1∶400 dilution), Anti-Klf4 (1∶400 dilution), Anti-Sox2 (1∶400 dilution), Anti-CD31 (1∶500 dilution), Anti-AQP-1 (1∶400 dilution), Anti-ZO-1 (1∶400 dilution), Anti-Nanog (1∶400 dilution) and Anti-SSEA4 (1∶400 dilution) for 60 min, and then with the secondary antibodies for 60 min at room temperature. The cells were rinsed with PBS twice for 3 min each time. Then, the samples were incubated in the moist chamber for 15 min with DAPI for nuclear stain. At last, the samples were washed again. The cells were examined by fluorescence microscope.

### Western blotting

The cell extracts were mixed with Laemmli buffer and boiled for 5 min. Proteins were separated for 1.5 h in 10% SDS-PAGE (Bio-Rad). Subsequently, proteins were transferred to nitrocellulose membranes (Amersham) using semidry transfer buffer (25 mM Tris, 150 mM glycin, 10% (v/v) methanol) and 3 mA/cm^2^ current for 45–50 min. Nitrocellulose membranes were blocked with 3% milk in TBS-T (0.1% Tween-20) for 1 h at room temperature. The primary antibody (Invitrogen) in blocking buffer was applied overnight at 4°C. After washing with PBS, the membranes were exposed to the secondary antibody for 1 h, and then detected by ECL technique (Amersham).

### Statistical analysis

The values were expressed as means ± SD from three to six samples. Statistical analyses were carried out using Student's t test and a one-way analysis of variance (SPSS 16.0, Inc., Chicago, IL, USA). Results of *p*<0.05 were considered statistically significant.

## Results

### The purification of reprogramming proteins and the identification of their binding activities with their target DNA sequences

**Figure 2:**
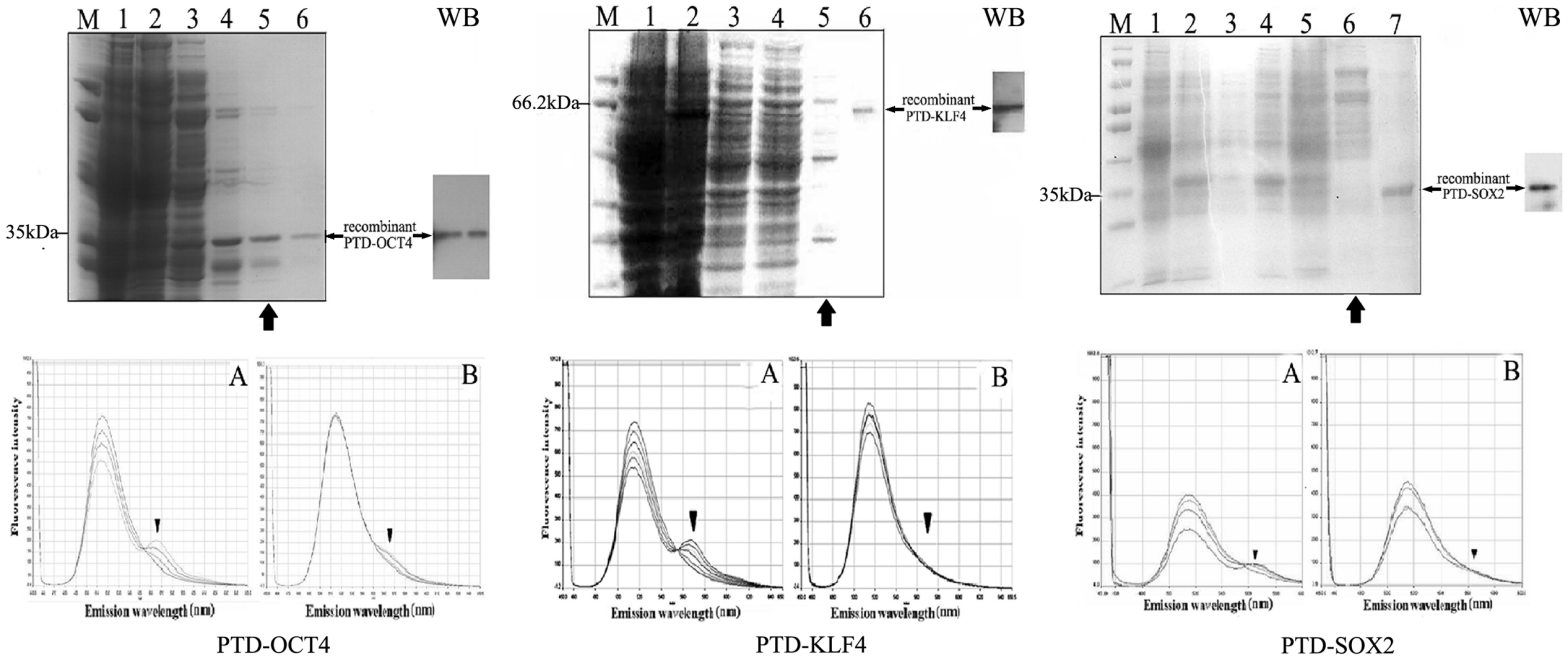
The purification and identification of reprogramming proteins. (Up) SDS-PAGE analysis displayed that fusion recombinant proteins of PTD-Oct4/Klf4/Sox2 were loaded onto a Ni affinity column and eluted with 60 mmol/L imidazole (indicated by large arrows). Small arrows revealed the identification of purified reprogramming proteins of PTD-Oct4 (35 kDa), PTD-Klf4 (66.2 bp) and PTD-Sox2 (35.8 kDa) by SDS-PAGE and western blotting (WB). (Down) There were significant FRET signals on 565 nm (PTD-Oct4), 570 nm (PTD-Klf4) and PTD-Sox2 (570 nm) (down A, indicated by arrow heads), while no FRET signal between reprogramming proteins and non-target sequence (down B).

The recombinant vectors of PKYB-PTD-Oct4/Klf4/Sox2-6His were successfully constructed. After they were transformed into ER2566, fusion PTD-Oct4, PTD-Klf4 and PTD-Sox2 were expressed and purified by Ni-affinity chromatography. The gradient concentration of imidazole was set to obtain the optimal elution concentration. SDS-PAGE analysis and western blotting identification displayed that 60 mmol/L imidazole elution could be used for the purification of PTD-Oct4 (35 kDa), PTD-Klf4 (66.2 kDa) and PTD-Sox2 (35.8 kDa) (Fig. 2 up). The fluorescence energy scanning of PTD-Oct4, PTD-Klf4 and PTD-Sox2 in FRET following the respective addition of the Oct4, Klf4 and Sox2 target sequences showed that the fluorescence emission intensity on 565 nm, 570 nm and 570 nm was increased following the addition of their target sequences (Fig. 2 down A), while there was no significant fluorescence emission intensity increase promoted by non-target DNA sequences (Fig. 2 down B). The result of FRET showed that the recombinant reprogramming proteins of PTD-Oct4, PTD-Klf4 and PTD-Sox2 had the specific activity to recognize and bind their target DNA sequences respectively.

### Characterization and differentiation of human ADSCs

**Figure 3:**
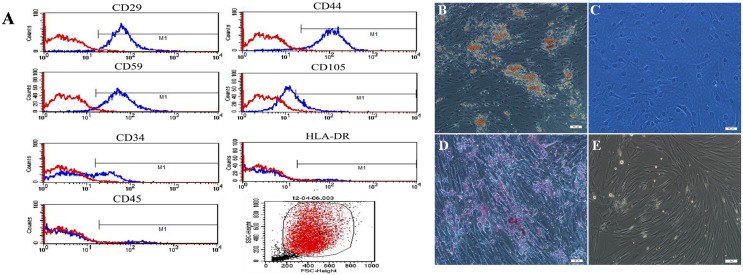
The characterization and differentiation of human ADSCs. (A) Flow cytometry analysis of surface phenotypes of human ADSCs. (B) The adipogenic differentiation of human ADSCs stained with Oil Red O and (C) negative control. (D) The osteogenic differentiation of human ADSCs stained Alizarin red and (E) negative control. Scale bars represent 100 µm.

Human ADSCs were isolated from human lipoaspirate tissue. A confluence of 80%–90% was reached after 1 week of culture. Flow cytometry analysis for the surface phenotypes of human ADSCs showed that primary hADSCs expressed MSC specific markers including CD29 (+), CD44 (+) and CD59 (+) but did not express CD45 (−) and HLA-DR (−). There were slight expressions of CD105 and CD34, which indicated the presence of endothelial cells and progenitors (Fig. 3A). hADSCs were spindle-shaped under phase contrast microscope. The adipogenic differentiation was detected by the formation of lipid droplets in cell cytoplasm stained with Oil Red O. Accumulation of lipid droplets in the cells after adipogenic differentiation of consecutive 2 weeks (Fig. 3B) while no lipid droplets in the negative control (Fig. 3C). Osteogenic differentiation was demonstrated by calcification areas shown by Alizarin red staining (Fig. 3D), in contrast, no calcification in the negative control (Fig. 3E).

### Primary test of reprogramming reagents PTD-OKS reprogramming proteins and small molecules on human ADSCs

#### The survival assay of human ADSCs treated with reprogramming reagents

**Figure 4:**
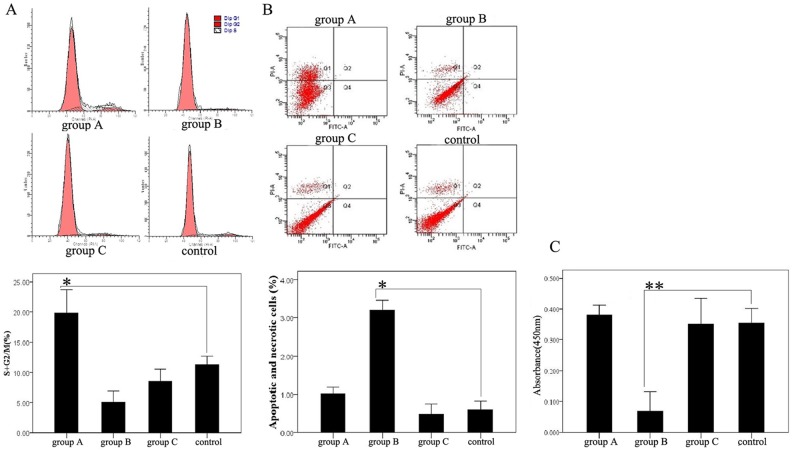
The survival assay of human ADSCs treated with reprogramming reagents. (A) Flow cytometric analysis of cell cycle of ADSCs treated with reprogramming reagents. (B) Flow cytometric analysis of apoptosis and necrosis of ADSCs treated with reprogramming reagents. The Q2 area represented cell necrosis, the Q3 area represented living cells and the Q4 area represented early apoptotic cells. (C) The proliferation in ADSCs treated with reprogramming reagents. Difference with P<0.05(*) was statistically significant.

In order to know whether or not PTD-OKS and small molecules had a cytotoxic effect, we first tested reprogramming reagents on the survival of human ADSCs. Human ADSCs cultured in DMEM containing 10% FBS were used as control group. Flow cytometric analysis of cell cycle distribution showed that the cell-cycle entrance of ADSCs treated with PTD-OKS (group A) was significantly higher than control group, while both group B and group C (PTD-OKS and small molecules) was obviously lower than control. The percentage of cells entering the S phase and G2/M phase was 19.80%±1.59% (group A), 5.06%±0.75% (group B), 8.54%±0.79% (group C) and 11.16%±1.6% (control) respectively (Fig. 4A). Annexin V expression and PI staining were analyzed by flow cytometry to detect apoptosis and necrosis in cultured ADSCs under various treatments. The apoptotic and necrotic cells in ADSCs of group B obviously increased, which was 3.2%±0.10%, while the percentages of apoptotic and necrotic cells were 1.02%±0.07% (group A), 0.45%±0.04% (group C) and 0.59%±0.09% (control) respectively (Fig. 4B). CCK-8 assay revealed that the proliferation of ADSCs in group B significantly lower than that in control. While the proliferation of ADSCs in group A and group C showed almost similar proliferation level as control (Fig. 4C).

#### The ability of the transduction of reprogramming proteins into ADSCs

**Figure 5:**
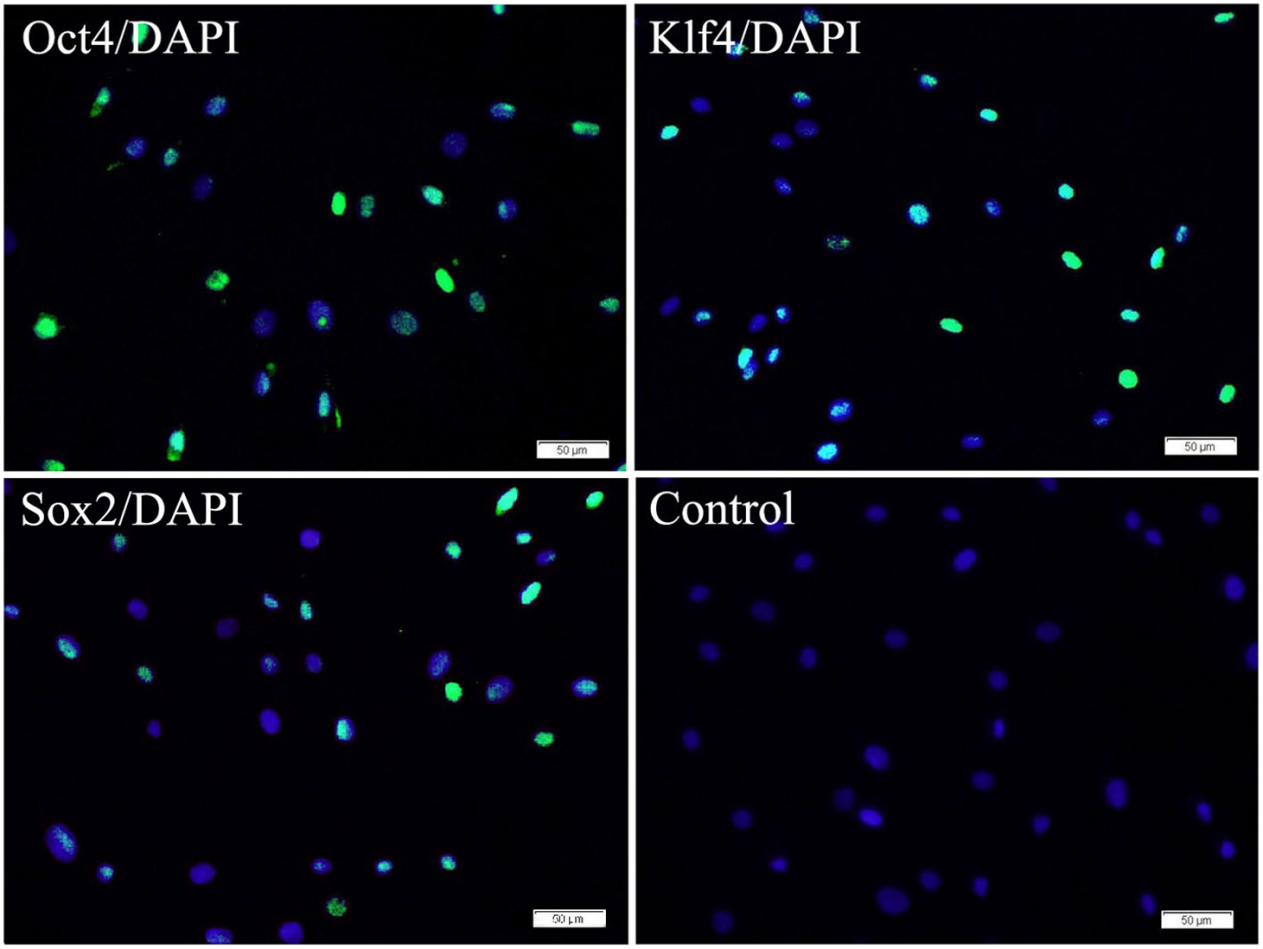
The transduction of reprogramming proteins into ADSCs by immunofluorescence staining. The nuclei of ADSCs (about 50% ∼60%) showed detectable fluorescence from specific antibodies of Oct4, Klf4 and Sox2 after ADSCs were transduced with reprogramming proteins (PTD-Oct4, PTD-Klf4 and PTD-Sox2) respectively for 4 h and then cultivated in conventional medium for 20 h. No fluorescent cells were observed when ADSCs were incubated with PBS in control. Scale bars represent 50 µm and DAPI for nuclear staining.

The ability of the recombinant reprogramming proteins (PTD-Oct4, PTD-Klf4 and PTD-Sox2) to penetrate into human ADSCs was analyzed by immunofluorescence staining. ADSCs were transduced with reprogramming proteins respectively for 4 h and then cultivated in conventional medium for 20 h. The cellular localization was visualized by immunofluorescence microscopy as shown in Fig. 5. The nuclei of about 50% ∼60% ADSCs showed detectable fluorescence. No fluorescent cells were observed when ADSCs were incubated with PBS in control.

#### The primary morphologic observation of human ADSCs treated with reprogramming reagents

**Figure 6:**
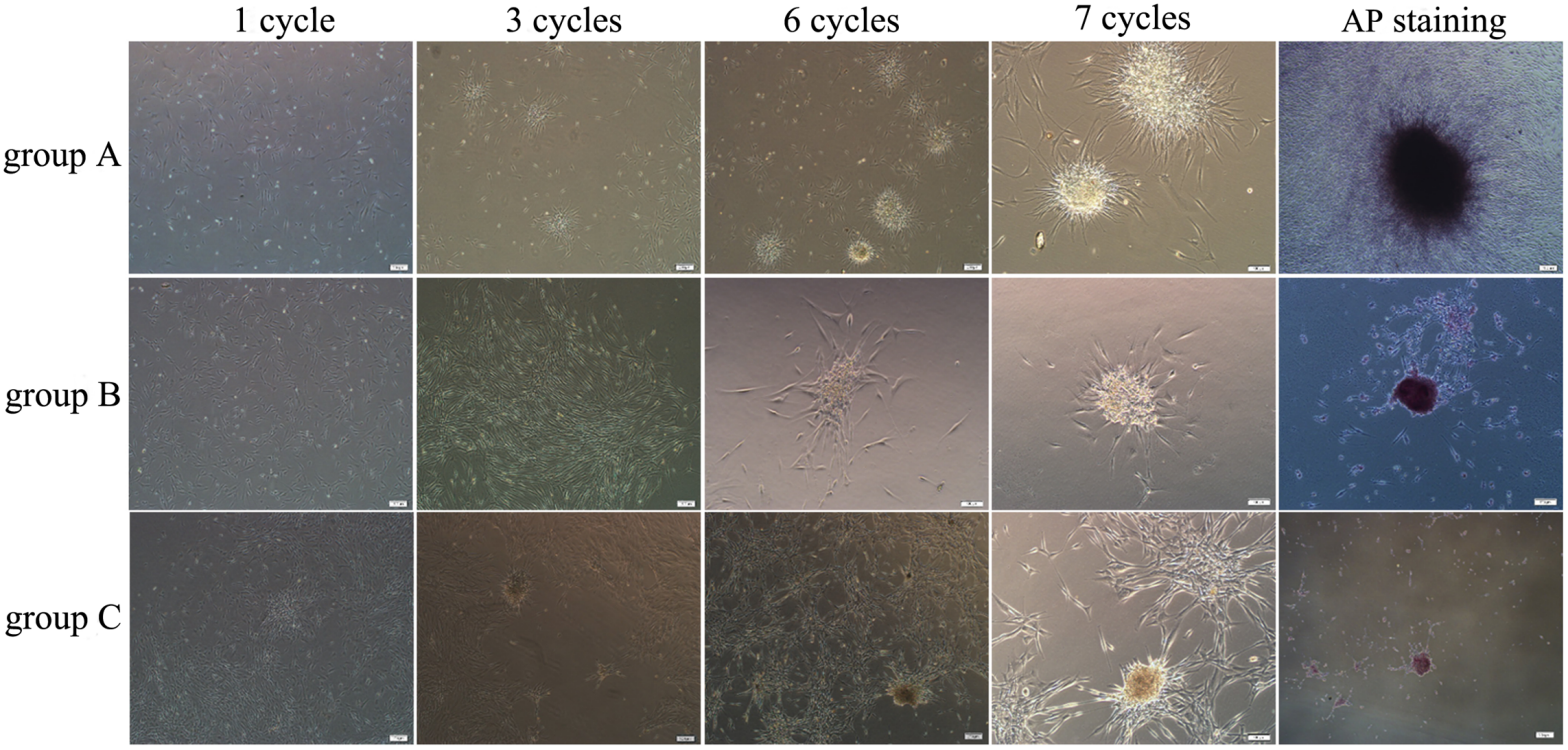
The cellular morphological conversion of human ADSCs after the treatment of reprogramming reagents. The adherent human ADSCs cultured after the treatments at 1, 3, 5 and 7 cycles in group A, group B and group C. ADSCs tended to aggregate growth at 5 cycles, and they displayed densely spheroids after 7 cycle treatment. ADSCs spheroids in all three groups were positive for AP staining. Scale bars represent 100 µm.

Primary human ADSCs were treated with reprogramming reagents in group A, group B and group C for 7 cycles. All ADSCs in these three groups showed the morphological changes of gradually decreased the adhesion on tissue culture plates and increased the aggregation among cells. ADSCs proliferated and displayed densely spheroids after 7 cycle treatment. ADSCs aggregated spheroids in these three groups were positive for AP staining (Fig. 6). However, ADSCs in control group always displayed spindle-shape cellular morphology while spheroid formation and AP staining was negative.

### Improved reprogramming effect on human ADSCs treated with modified reagents and SMG culture

**Figure 7:**
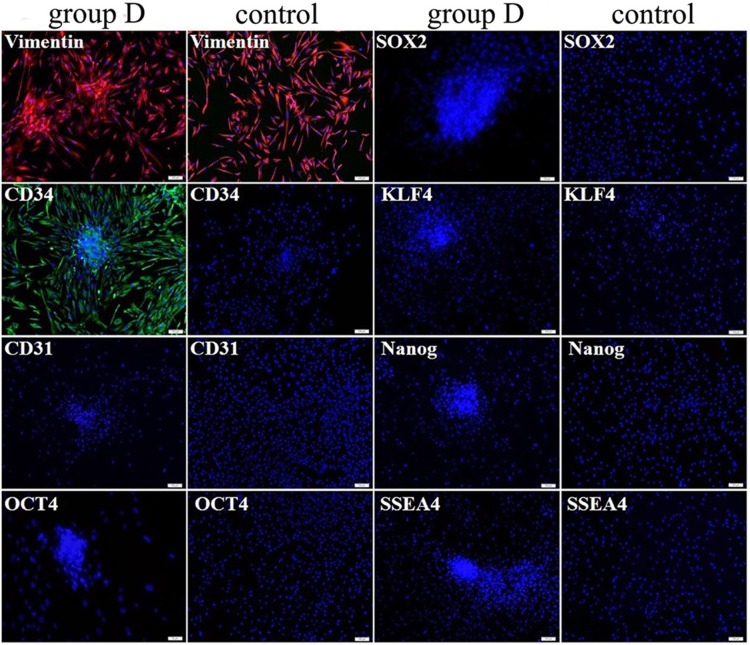
The immunofluorescence staining of human ADSCs after the treatment of modified PTD-OKS proteins supplemented with purmorphamine. ADSCs spheroids in group D were positively stained for vimentin and CD34 by immunofluorescence staining while they did not detect CD31 and undifferentiated stem cell markers expression. ADSCs in control group only showed positive staining for vimentin. Scale bars represent 100 µm and DAPI for nuclear staining.

In group D, primary ADSCs were easier and earlier to form aggregation after the treatment of modified PTD-OKS proteins supplemented with purmorphamine than other groups. The cellular aggregated spheroids were also positive for AP staining. Immunofluorescence identification revealed that vimentin and CD34 were expressed in ADSCs spheroids after 7 cycle treatment of PTD-OKS and purmorphamine, whereas negatively stained for CD31 and undifferentiated stem cell markers, such as Oct4, Sox2, Klf4, SSEA4 and Nanog. ADSCs in control group only showed positive staining for vimentin (Fig. 7).

**Figure 8:**
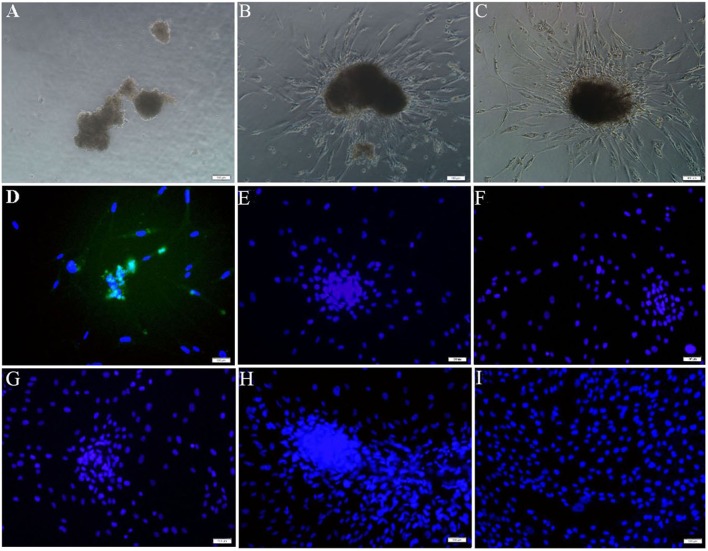
The conversion of human ADSCs after the treatment of modified PTD-OKS proteins supplemented with purmorphamine and microgravity culture. (A) ADSCs formed big and dense aggregations in group E after microgravity culture on day 5. (B) These ADSCs spheroids readily attached to the surface of plates after they were re-plated onto the adherent culture plates on day 5. (C) The spheroids generated cells that eventually repopulated as a confluent monolayer on day 7. These adherent ADSCs spheroids in group E positively expressed Nanog (D), while negatively expressed Oct4 (E), Sox2 (F) and Klf4 (G) by immunofluorescence staining on day 9. ADSCs spheroids in group D (H) and cells in control group (I) did not express Nanog by immunofluorescence staining. Scale bars represent 100 µm and DAPI for nuclear staining (D–I).

**Figure 9:**
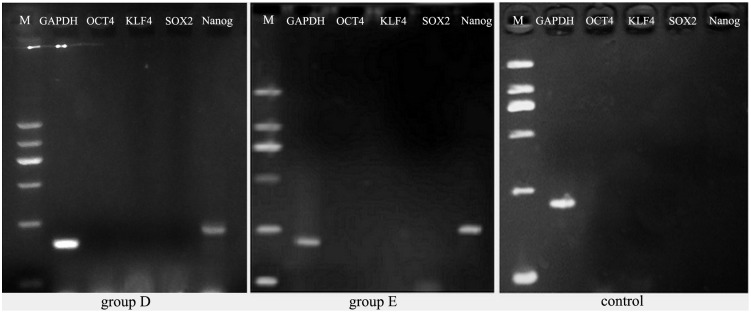
RT-PCR analysis for the undifferentiated gene expressions of human ADSCs after reprogramming treatment. The gene expressions of Nanog of human ADSCs spheroids after 7 cycle treatment of PTD-OKS and purmorphamine in group D and after microgravity culture in group E was positively displayed. However, ADSCs in control group did not express Nanog gene. The undifferentiated gene expressions of Oct4, Sox2, Klf4 were negative in all ADSCs and GAPDH were expressed in all ADSCs.

In group E, ADSCs after 7 cycle treatment of PTD-OKS and purmorphamine were cultured in simulated microgravity system. When ADSCs cultured under SMG condition for five days, small spheroids grew and enlarged. Some spheroids fused each other to form big and dense aggregations (Fig. 8A). These ADSCs spheroids readily attached to the surface of plates after they were re-plated onto the adherent culture plates (Fig. 8B). Following attachment, the 3-D spheroids generated cells that eventually repopulated as a confluent monolayer (Fig. 8C). Immunofluorescence staining showed that Nanog was positively expressed in these adherent ADSCs spheroids (Fig. 8D), while negatively expressed Oct4 (Fig. 8E), Sox2 (Fig. 8F) and Klf4 (Fig. 8G). ADSCs did not express Nanog in static group D (Fig. 8H) and in control group by immunofluorescence staining (Fig. 8I). RT-PCR analysis showed that the gene transcript of Nanog in human ADSCs spheroids after 7 cycle treatment of PTD-OKS and purmorphamine in group D (Fig. 9A) and after microgravity culture in group E (Fig. 9B) was positively expressed. The results showed that SMG culture condition were able to promote the stemness reprogramming for human ADSCs. However, ADSCs after conventional culture in control group did not express Nanog gene (Fig. 9C). The undifferentiated gene expressions of Oct4, Sox2, Klf4 were negative in all ADSCs and GAPDH were expressed in all ADSCs.

### The conversion of human ADSCs after co-cultured with corneal cells and tissue

**Figure 10:**
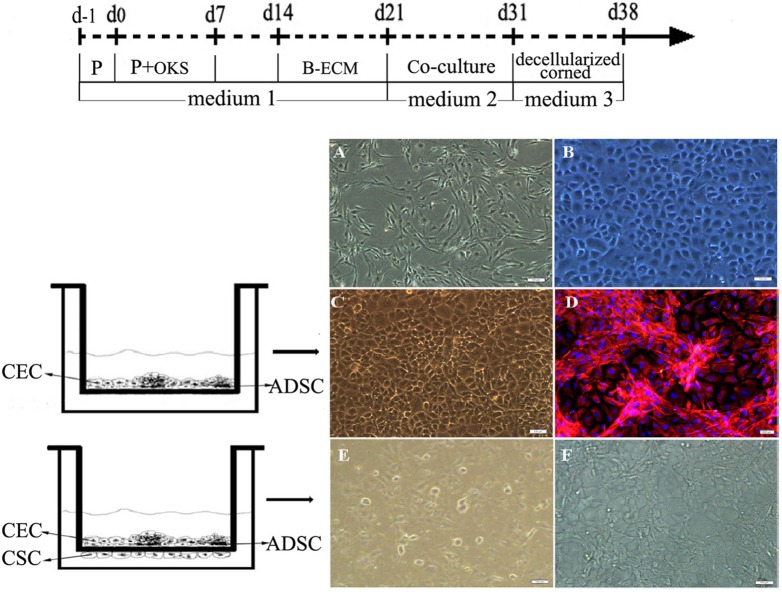
The conversion of human ADSCs after co-cultured with corneal cells and tissue. (Up A) The conventional cultured human ADSCs displayed spindle shape. (B) The primary cultured rabbit CECs showed hexagonal cobblestone shape. (C) Human ADSC mixed co-culture with rabbit CECs treated with MMC in plates and (D) immunofluorescence staining was positive for vimentin mainly in ADSCs. (E) ADSCs co-culture with both of R-CECs and R-CSCs showed polygonal tendency. (F) ADSCs on decellularized corneas after the treatment of sequential direct reprogramming and biomimetic platforms displayed polygonal morphology. (Up) The schematic illustrated sequential non-genetic direct reprogramming for ADSCs into CEC-like cells. Scale bars represent 100 µm (A–F) and DAPI for nuclear staining (D).

**Figure 11:**
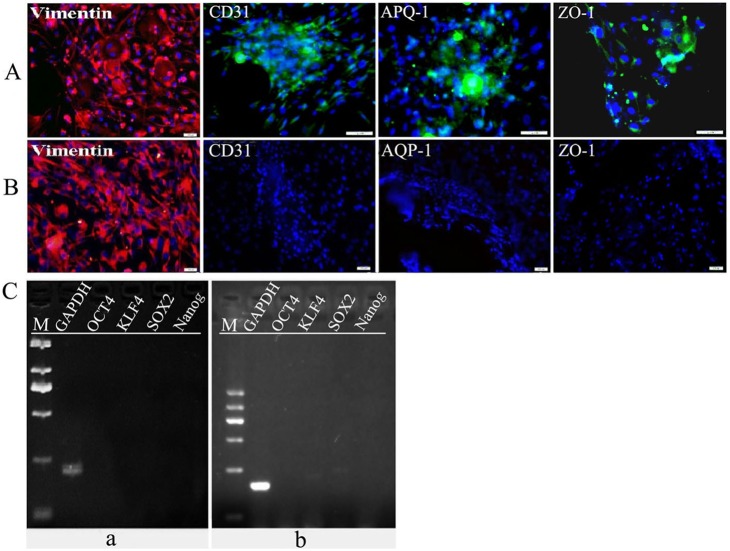
The immunofluorescence staining and RT-PCR analysis of human ADSCs after sequential non-genetic direct reprogramming and co-culture. (A) Human ADSCs on decellularized corneas after sequential non-genetic direct reprogramming with co-culture treatments of both of R-CECs and R-CSCs were obviously positive staining for vimentin and weakly expressed CD31, AQP-1 and ZO-1. (B) ADSCs on decellularized corneas after sequential non-genetic direct reprogramming without co-culture treatments were positive staining for vimentin but negative for CD31, AQP-1 and ZO-1. (C) The undifferentiated gene transcripts of Oct4, Sox2, Klf4 and Nanog could not be detected in ADSCs on decellularized corneas after sequential non-genetic direct reprogramming with (a) or without (b) co-culture treatments. Scale bars represent 100 µm and DAPI for nuclear staining (A, B).

The conventional cultured human ADSCs displayed spindle shape (Fig. 10A) while primary cultured rabbit CECs showed hexagonal cobblestone shape (Fig. 10B). When human ADSCs mixed co-culture with R-CECs treated with MMC in plates, fibroblast-like cells and polygonal cells could well survive together (Fig. 10C). ADSCs tended to aggregate and interconnect to form reticular morphology while CECs were inclined to grow as flat monolayer. Immunofluorescence staining was positive for vimentin mainly in ADSCs (Fig. 10D). ADSCs co-cultureed with both of R-CECs and R-CSCs showed polygonal tendency (Fig. 10E). ADSCs after sequential non-genetic reprogramming treatment and co-culture with both of R-CECs and R-CSCs could well grow on the decellularized corneas and displayed polygonal morphology (Fig. 10F). The schematic illustration of sequential non-genetic direct reprogramming and biomimetic platforms in this preliminary study for ADSCs into CEC-like cells was shown in Fig. 10 (up). Immunofluorescence assay revealed that human ADSCs on decellularized corneas after such sequential non-genetic direct reprogramming with co-culture treatments of both of R-CECs and R-CSCs were obviously positive staining for vimentin and weakly expressed CD31, AQP-1 and ZO-1 (Fig. 11A). However, ADSCs on decellularized corneas after sequential non-genetic direct reprogramming without co-culture treatments were positive staining for vimentin but negative for CD31, AQP-1 and ZO-1 (Fig. 11B). RT-PCR analysis showed that the undifferentiated gene transcripts of Oct4, Sox2, Klf4 and Nanog could not be detected in ADSCs on decellularized corneas after sequential non-genetic direct reprogramming with or without co-culture treatments (Fig. 11C).

## Discussion

Yamanaka factors are able to reprogram somatic cells to become iPSCs. But generation of iPSCs and directed differentiation from iPSCs are laborious and inefficient. At the same time, iPSCs always present the problems on safety, genetic and epigenetic aberrations. Somatic cell reprogramming has recently prompted the study on direct lineage conversion between two mature cells. Such direct reprogramming can be generally accomplished on a short timescale ranging, more efficient and safe [4], [32]. Several attempts show that a combination of Yamanaka factors with specific developmental and physiological cues can generate plastic reprogramming intermediate state and subsequent induction of the fates of target cells, which effectively make lineage conversion between two differentiated somatic cells [8]–[9], [33]–[34].

Somatic stem- and progenitor-cells in the adults share some features with pluripotent stem cells, so these cells are efficient source of iPSCs. For example, immature cell populations of the hematopoietic lineage in general give rise to iPS cells at higher efficiencies than terminally differentiated cell types [35]. Using human ADSCs as donor cells for reprogramming has several advantages. First, the isolation and culture of ADSCs are relatively simple, fast, and safe. Second, ADSCs can be readily obtained from adult humans in large quantities and represent an ideal autologous source of cells for reprogramming. Third, ADSCs express AP activities and have the high endogenous expression level of Klf4, Klf2, Klf5, Esrrb, and c-Myc, which make ADSCs more plastic and fewer barriers for reprogramming. Many studies revealed that the generation of iPSCs from human ADSCs with a faster speed and higher efficiency than adult human fibroblasts using Yamanaka factors [36], [37]. In this study, ADSCs could be easily isolated by collagenase digestion from human lipoaspirate tissues. ADSCs were positive for CD29, CD44 and CD59 and negative for CD45 and HLA-DR, which were characteristic expressions of MSCs [38], [39]. Primary ADSCs also partly expressed CD34 and CD105, which showed that these ADSCs were comprised of heterogeneous cell populations. CD34 was quiescence stem cell and endothelial cell marker [40]. Usually, CD105 expression was relatively low on the freshly isolated ADSCs. The expression level of CD34 decreased while that of CD105 increased for a period of time of ADSCs culture [41].

Somatic cell reprogramming techniques involving genome integration and genetic manipulation are usually complicated by the potential risks, such as insertional mutations of host genome, tumorigenesis and so on. For example, retroviral expression of two reprogramming factors (c-Myc and Klf4) and one chondrogenic factor (Sox9) induced chondrogenic cells directly from adult dermal fibroblast cultures. However, some induced cell lines formed tumors when subcutaneously injected into nude mice [34]. Therefore, for the sake of safe clinical application, nonintegrating or non-DNA overexpression strategies for iPSC generation or lineage conversion should be applied [42]. Recently, several approaches have been developed to generate transgene-free or integration-free cell reprogramming [43]. One of safe approaches for cell reprogramming is chemical genetics that uses small modulators involved in the regulation of cell states, which is faster, reversible, and more controllable [17]. Another rational approach to achieve non-genetic reprogramming cells is the uses of reprogramming proteins with cell-penetrating peptides (CPPs) or protein transduction domains (PTDs) [44]. The combinative uses of small molecule VPA regimen and recombinant proteins with CPPs or PTDs showed significantly higher reprogramming efficiency than their separate application [13], [18]. We found that the specific binding capacity of PTD-Oct4, PTD-Klf4 and PTD-Sox2 reprogramming proteins with their target DNA sequences were about 28.3%, 40.86% and 22.29% respectively. Using these reprogramming proteins alone or supplemented with purmorphamine, RG108 and other small molecules, ADSCs easily formed aggregated growth and were positive for AP staining. Especially, we found that PTD-OKS proteins supplemented with purmorphamine displayed higher cell survival and lower apoptosis than other reprogramming reagents. ADSCs were positive for stem cell and endothelial cell marker CD34 by immunofluorescence staining and gene expressions of undifferentiated marker Nanog after modified procedure of the treatment of PTD-OKS proteins supplemented with purmorphamine. It was reported that Bmi1 was able to replace Sox2, Klf4, or C-Myc in inducing Nanog-positive colonies that resembled ESCs. The activation of sonic hedgehog (Shh) signaling by purmorphamine could compensate for the effects of Bmi1. Purmorphamine together with Oct4 is sufficient for the generation of iPSCs from mouse embryonic fibroblasts (MEFs) and adult fibroblasts [3]. Purmorphamine not only stimulates the Shh pathway but also activates Shh target gene transcription through the protein Smo [45]. MEFs could also be reprogrammed to pluripotency by combinations of purmorphamine and 2i/LIF (MEK/ERK and GSK3 pathway inhibitors in the presence of leukemia inhibitory factor) [46]. There were several reports published on the effects of purmorphamine on human mesenchymal stem cells (hMSCs), yet their results and conclusions were quite diversified and contradictory. It was demonstrated that purmorphamine increased the expression of a panel of genes related to osteoblast phenotype development in hMSCs [47]. Purmorphamine activated hedgehog signaling pathway, inducing osteogenesis in the rodent cell line. [48] However, it was observed that gene expression of RUNX2, osteopontin, osteoprotegerin, and osteonectin were inhibited after hedgehog pathway activation in ADSCs and hMSCs [49]. Oliveira et al. considered that such discrepancy could be related to distinct osteoblast differentiation status of cultures. The effect of activation of Shh signaling on osteoblast differentiation of hMSCs was closely related to the culture conditions and the stage of osteoblast maturation [47]. Purmorphamine caused an increase of cell proliferation in ADSCs, but matrix mineralization was absent, when ADSCs cultured with ascorbic acid. Comparatively, cultures treated with dexamethasone, purmorphamine caused a decrease of cell proliferation and formation of sporadic mineral deposition in ADSCs [50]. In our culture conditions, purmorphamine supplemented with PTD-OKS proteins displayed the promotion of ADSCs spherical growth, positively expressed Nanog and CD34, while did not show the characteristics and effect on osteoblast differentiation in ADSCs. The results confirmed that purmorphamine was able to promote some stemness reprogramming for human ADSCs. It is well known that purmorphamine is Shh activator and can compensate for the effects of Bmi1. Bmi1 was able to replace Sox2, Klf4, or C-Myc in inducing Nanog-positive colonies [3]. Furthermore, we also tried SMG culture for reprogramming. We found that microgravity culture without the addition of microcarriers was able to intensify the reprogramming effect. Such dynamic SMG condition promoted both phenotype and gene expression of Nanog in human ADSCs after the treatment of PTD-OKS proteins supplemented with purmorphamine. These results suggest for the first time that SMG rotary cell culture system is able to promote the stemness reprogramming for human ADSCs and can be used as a non-genetic means to enhance direct reprogramming. Recent report indicated that SMG rotary cell culture could provide efficient gas transfer, uniform and low-shear environments for ESCs and iPSCs growth and differentiation [51]. Rat MSCs cultured under SMG showed undifferentiated state such as dome-like shape and the expression of Oct-4 mRNA [52].

The contact co-culture was an effective means for the differentiation of stem cells. Usually, stem cells co-cultured with target cells or stromal cells could well promote the differentiated conversion. For example, the orbital fat-derived stem cells (OFSCs) converted to express cytokeratin-19 and cytokeratin-3 after mixture co-culture with corneal epithelial cells, which demonstrated that direct contact with target cells was essential for OFSCs to commit to corneal epithelial cells. [53] Perrier et al. reported that using stromal cell–derived inducing activity (SDIA) culture method, co-culture of hES cells on MS5 stroma yielded highly efficient differentiation into neuroepithelial structures. [54] In this study, we tried human ADSCs co-cultured with target cells (rabbit CECs) in direct mixture and transwell contact with stromal cells of rabbit CSCs. We found that cells well survived together under such contact co-culture model. The insert porous membrane of transwell acting as a physical barrier prevented cells contamination but can induce direct interaction among cells. ADSCs revealed polygonal morphology from spindle shape after our sequential non-genetic reprogramming treatment, co-culture with R-CECs and seeded onto decellularized corneal stroma. At the same time, induced cells converted to weakly express CD31, AQP-1 and ZO-1, which were endothelial markers [55], [56]. Wu’s laboratory demonstrated that adult rat CEC-derived conditioned medium could induce rat neural crest cells (NCCs) to differentiate into polygonal CEC-like cells. These CEC-like cells seeded onto decellularized corneal stroma showed positive immunofluorescence stains of ZO-1 and Na^+^/K^+^ ATPase. Their experiment revealed that paracrine factors from adult CEC-derived conditioned medium (culture supernatant) and acellular corneal ECM acted upon the NCCs differentiation and promoted the induced cells to form premature and mature CEC-like cells [57]. Our study partly obtained similar results. We speculate that sufficient CEC differentiation needs more complete induction procedure, media and microenvironment, which should comply with the changes of time and track of corneal endothelial development.

Biomimetic platforms in vitro replicate the context of target organ or tissue in vivo, which recapitulate the microenvironments associated with tissue development, physiology and regeneration. The biomimetic conditions in combination from 3-D environment of bioreactors, cell-cell contacts of co-culture, and cell-matrix interactions of decellularized native ECM can supply molecular and physical signals to guide cell reprogramming and differentiation paths [25], [58]. In this preliminary study of non-genetic direct reprogramming, we found that human ADSCs come out some undifferentiated states when treated with Oct4/Klf4/Sox2 proteins supplemented with small molecule purmorphamine. The biomimetic platforms such as SMG bioreactor condition, co-culture, and decellularized corneal ECM promoted the direct reprogramming effects. For the safe consideration, we avoided genome integration and bypassed the pluripotent state to activate ADSCs with proteins of Oct4/Klf4/Sox2 and small molecule. Therefore, we avoided complications associated with the use of genetic manipulation, onco-protein c-Myc and iPSCs in this study. As a step toward better CECs differentiated effect, our further study will add the use of the signaling cues of CECs developmental process. It was reported that CECs could be efficiently induced from human cornea-derived precursors (COPs) when mimicked developmental process from the NCCs to CECs in vitro [59]. In fact, biomimetic approach also included the regulatory signals during native development and regeneration to direct the differentiation and functional assembly of stem cells [25]. Moreover, for the future clinical application, xeno-free cell culture conditions should be used to minimize the risk of transmitting disease and immunological reactions [60]. So, we should choose human CECs co-cultured with human ADSCs and animal-free serum replacements. Recent report indicates that direct reprogramming offers a potentially very attractive alternative to the rather circuitous iPSC methodology for the generation of autologous tissue. How to increase protocol effectiveness would be critical for adaptation to the human system and eventual therapeutic use [6]. We believe that the optimal non-genetic direct reprogramming and biomimetic platforms to induce mature human CECs will be discovered in the near future, which will be beneficial for potential CECs transplantation and helpful for the therapy of reduced visual acuity due to CECs deficiency.
